# Nutritional risk and associated factors of adult in-patients at a teaching hospital in the Copperbelt province in Zambia; a hospital-based cross-sectional study

**DOI:** 10.1186/s40795-018-0249-4

**Published:** 2018-12-06

**Authors:** Nixon Miyoba, Joseph Musowoya, Emily Mwanza, Angel Malama, Nyati Murambiwa, Irene Ogada, Macriveness Njobvu, Doris Liswaniso

**Affiliations:** 1 Kitwe Teaching Hospital, Kitwe, Zambia; 2 Ndola Teaching Hospital, Ndola, Zambia; 3 The University of Zambia, Lusaka, Zambia; 4 Saint Francis Xavier University, Antigonish, Canada; 5 Ministry of Agriculture and Cooperatives, Kalulushi, Zambia

**Keywords:** Adult inpatients, Teaching hospital, Nutritional risk, Malnutrition, Undernutrition

## Abstract

**Background:**

Nutritional risk and undernutrition are common problems among medical and surgical patients. In hospital, malnutrition is frequently under-diagnosed and untreated thereby contributing to morbidity and mortality. The purpose of this study was to determine the prevalence of nutritional risk among adult inpatients at a teaching hospital in Zambia. In addition, the study sought to establish factors associated with nutritional risk.

**Methods:**

A hospital-based cross-sectional study comprising of 186 consecutive in-patients aged 18–64 years admitted in medical and surgical wards was conducted at a teaching hospital. Out of one hundred and ninety eight (198) patients eligible to participate, complete data were collected from 186, representing a response rate of 93.9%. The Malnutrition Universal Screening Tool was used to collect data over a period of six months. Evaluated patients were dichotomized into no risk and nutritional risk. Binary logistic regression was performed to identify variables associated with nutritional risk.

**Results:**

The mean age of adult in-patients was 40.72 ± 14.4 years. Majority of the patients were male (61.8%), while 38.2% were female. Results indicate that 59.7% of hospitalized patients were at nutritional risk. Vomiting, weakness, appetite decrease, dysphagia and weight loss were significantly associated (*p* = 0.019, *p* = 0.008, *p* < 0.001, *p* = 0.007, and *p* < 0.001 respectively) with nutritional risk. However, weight loss and appetite decrease were the most significant factors associated with nutritional risk (OR = 50.16, 95% CI = 5.75–36.70, *p* < 0.001 and OR = 28.06, 95% CI =1.49–8.12, *p* = < 0.001 respectively).

**Conclusion:**

Findings of our study suggest that close to 60% of adult inpatients at the teaching hospital were at nutritional risk. Nutritional risk is an issue of major concern at the teaching hospital and is associated with a number of variables. Identification of nutritional risk using Malnutrition Universal Screening Tool among adult inpatients is feasible in resource-poor settings like ours.

**Electronic supplementary material:**

The online version of this article (10.1186/s40795-018-0249-4) contains supplementary material, which is available to authorized users.

## Background

Nutritional risk is an important indicator used to predict the probability of clinical outcomes related to nutritional factors [1]. Upon admission to hospital, all patients should undergo routine nutritional risk screening [2]. Policy guidelines and protocols for identifying patients at nutritional risk should be in place in hospitals [1]. Screening is the initial step in identifying patients at nutritional risk [1, 2]. It is a simple and rapid procedure used by health care professionals on first contact with patients [2]. Through screening, nutritional problems can be detected early and appropriate nutritional interventions developed for patients at significant risk [1].

Globally, a significant proportion (15–70%) of hospitalized patients are undernourished [3, 4]. In the United Kingdom, data from clinical settings indicates that 16–21% of patients are at nutritional risk [5, 6]. The findings suggest that patients at medium and high risk of malnutrition experience frequent readmissions and stay longer in hospitals [5, 6]. A study conducted in Denmark reported that 14 out of 740 patients were at risk of malnutrition [1]. The prevalence of nutritional risk in a multicenter study conducted in Beijing Teaching Hospitals in China was estimated at 27.3% [7]. However, only 24.9% of the patients at nutritional risk received nutritional support [7].

Several studies have reported that undernutrition and nutritional risk are common problems among medical and surgical patients [8–11]. In hospital, malnutrition is frequently under-diagnosed and untreated thereby causing various adverse effects [12]. Malnutrition remains undiagnosed in close to 70% of hospitalized patients [3]. An estimated 80% of malnourished patients are discharged without receiving any nutritional support [3, 7]. Patients at nutritional risk have higher rates of infection, complications and suffer from organ failure [13]. Further, malnutrition results in delayed wound healing, suboptimal response to treatment, mortality and longer hospital stay [13–16]. Timely identification and management of malnutrition is required as the condition is not only a threat to a patients’ health but also increases healthcare costs [17]. In order to mitigate the negative outcomes of malnutrition, the condition should be correctly identified and treatment of patients at nutritional risk prioritized [18].

In sub-Saharan Africa, there is limited literature about nutritional risk of hospitalized patients. In Zambia, there are no guidelines recommending a nutritional screening tool for use among adult in-patients. Further, there is paucity of literature in hospitals about the nutritional risk of in-patients making it difficult for health workers to prioritize treatment of patients at high risk. At the teaching hospital, there are no protocols for identification of in-patients at nutritional risk. It is against this background that this study has been conceptualized to provide baseline information on nutritional risk and associated factors among adult inpatients at the teaching hospital.

## Methods

### Research design, period and location

A hospital-based cross-sectional design was adopted for this study. The design was appropriate as data was collected from consecutive patients at one point in time [19]. The study was carried out from May to November 2017 at a teaching hospital, in Zambia. The teaching hospital has a bed capacity of 664 and is located in Kitwe District on the Copperbelt province [20]. The hospital offers tertiary health care services to the population of Kitwe, other towns on the Copperbelt province, and also provides referral services to patients from Luapula, Northern and North-Western provinces [20].

### Study population

The study targeted adult in-patients (18–64 years). Adult in-patients admitted to medical and surgical wards and able to communicate were included in the study. The study excluded critically ill and cognitively impaired in-patients, those admitted in obstetric and gynaecology wards and those who declined to participate.

### Sampling techniques and sample size

The teaching hospital was purposively sampled because it is the only teaching hospital offering tertiary health care services in Kitwe District [20, 21]. Medical and surgical wards were purposively sampled because nutritional risk is reported to be a significant problem among patients admitted in those wards [8–11]. Consecutive sampling was used to include all patients who met the inclusion criteria and consented to participate in the current study [7].

### Research instruments

Identification of nutritional risk requires the use of validated and ease-to-use screening tools [22, 23]. In clinical settings, several nutrition screening tools are in use, each presenting variations in variability, reliability and acceptability [24, 25]. There is no tool that is universally accepted for identifying nutritional risk [26]. However, reliability and validity are essential criteria when deciding on a screening tool to use [25]. Malnutrition Universal Screening Tool (MUST) used in similar studies was employed in data collection [2, 7, 27]. MUST was designed to identify patients at nutritional risk and is recommended for use among adult inpatients [2]. The tool has been found to be rapid, reproducible and internally consistent. There are three variables of the MUST: Body Mass Index (BMI), unintentional weight loss and disease effect [28]. Each variable is scored as 0, 1, or 2 and patients are classified into three categories of low risk (0), moderate risk (1) and high risk (2) [2]. Evaluated patients were dichotomized into no risk (low risk) and nutritional risk (moderate and high risk) [28]. Validity was ensured by use of an already validated questionnaire with elements of MUST incorporated into the questionnaire [27]. The tools were also pre-tested. During the pre-test, the test-retest method was used to establish reliability of the questionnaire. Data was collected twice at an interval of 3 days from 19 patients. A correlation coefficient of 0.79 (CI: 0.67–0.85) was computed from the two sets of data and found to be adequate [29] Additional file 1.

### Data collection procedures

Names of admitted adult inpatients were obtained from admission registers in each ward. The investigator in consultation with sisters-in-charge of each ward generated a list of all eligible patients. Each patient was given a comprehensive explanation of the nature and purpose of the study. Voluntary informed consent was sought from participating patients prior to data collection.Guided by a structured questionnaire used in similar studies, face-to-face interviews were conducted in the local language with each patient on the ward [2, 27, 28]. Data on medical and demographic characteristics of patients was obtained from patient’s files and recorded in the questionnaires. Additional information on other medical aspects such as diarrhoea, vomiting, dysphagia, appetite decrease, extent of weight loss and food intake was verbally solicited from patients and/or their bedsidders. Standard methods were employed by the investigator to measure weight and height [30]. The measurements were taken in the morning before meals. The weight of the patients was measured while wearing light clothing using the same clinical scale and recorded to the nearest 0.1 kg. Scales were calibrated at the beginning of the study. Height was estimated to the nearest 0.1 cm using ulna length. Nutritional status data was enhanced by including information on mid-upper arm circumference and physical assessment [30]. At the end of each working day, MUST of all patients was computed using standard procedures and recorded by the investigators as: low risk (0), moderate risk (1) and high risk (2) [2]. Data was collected using the same procedure from consecutive patients for a period of six months (5/5/2017 to 10/11/2017).

### Statistical analyses

Prior to data entry, completed questionnaires were checked daily for accuracy and consistency. Statistical Package for Social Sciences (SPSS version 21.0) was used to analyze data. Descriptive statistics in terms of means, frequencies, percentages and standard deviation were generated. Test of quantitative variables for normal distribution was done using the Kolmogorov-Smirnoff test, as appropriate. Binary multivariate logistic regression was performed to determine the influence of selected variables on nutritional risk. A *p*-value of less than 0.05 was considered statistically significant.

## Results

Out of one hundred and ninety eight (198) patients eligible to participate, data was collected from 186 (115 male, 71 female) representing a response rate of 93.9%. The mean age of the study population was 40.72(14.4) years (Table 1).

**Table 1:** Selected characteristics of adult in-patients

Participant characteristics	Categories	*N* = 186 n	%
Sex	Male	115	61.8
	Female	71	38.2
Readmission	Yes	79	42.5
	No	107	57.5
Diarrhoea	Yes	20	10.8
	No	166	89.2
Vomiting	Yes	41	22.0
	No	145	78.0
Weakness	Yes	89	47.8
	No	97	52.2
Appetite decrease	Yes	96	51.6
	No	90	48.4
Dysphagia	Yes	38	20.4
Weight loss	No	148	79.6
	Yes	75	40.3
	No	111	59.7

In this study, mid-upper arm circumference (MUAC) of all patients was evaluated. The mean MUAC of the patients was 24.01(4.92) cm, ranging from 14.5 cm to 41.2 cm. The mean Body Mass Index of the patients was 22.65(2.4) Kg/m^2^. Patients’ physical assessment revealed that 59(31.7%) were moderately wasted and 19(10.2%) were severely wasted. Nutritional risk was determined by Malnutrition Universal Screening Tool (MUST) criteria. Results indicate that majority of the patients 83(44.6%) were at moderate risk, while 28(15.1%) were at high nutritional risk (Table 2).

**Table 2:** Classification of nutritional risk based on MUST criteria and reorganized categories of nutritional risk is according to Velasco 2011 (Moderate and high risk = nutritional risk, low risk = no risk)

Nutritional risk classification	*N* = 186 n	%
Nutritional risk		
Low risk	75	40.3
Moderate risk	83	44.6
High risk	28	15.1
Dichotomized nutritional risk		
No risk	75	40.3
Nutritional risk	111	59.7

As depicted in Table 2, evaluated patients were dichotomized into nutritional risk (moderate and high risk) and no risk (low risk). Results indicate that 59.7% of hospitalized patients were at nutritional risk.

Table 3 shows the characteristics/aspects associated with nutritional risk. The study reveals that weight loss and appetite decrease were the most significant factors associated with nutritional risk (OR = 50.16, 95% CI = 5.75–36.70, *p* < 0.001 and OR = 28.06, 95% CI =1.49–8.12, *p* < 0.001 respectively).

**Table 3:** Characteristics associated with nutritional risk after binary logistic regression with OR (odds ratio) and 95% confidence interval (95% CI)

Characteristics	OR	95% CI	*p*-value
Sex	0.65	(0.28–1.38)	0.419
Readmission	1.36	(0.59–2.98)	0.244
Diarrhoea	3.85	(0.46–2.98)	0.050
Vomiting	5.55	(0.70–5.79)	0.019
Weakness	7.07	(0.36–2.04)	0.008
Appetite decrease	28.06	(1.49–8.12)	< 0.001
Dysphagia	7.37	(0.42–4.05)	0.007
Weight loss	50.16	(5.75–36.70)	< 0.001

## Discussion

The findings of the present study reveal that 59.7% of adult in-patients were at nutritional risk. This result is consistent with that of a study conducted in Brazil which estimated the prevalence of nutritional risk at 60.7% [27]. In that study, data was collected using a structured questionnaire recommended by American Dietetic Association (ADA) [27]. A cross-sectional study in Danish hospitals indicated that close to 40% of patients were nutritionally at risk [31]. Another study conducted in three hospitals reported that 22.0% of inpatients were at nutritional risk [1]. Using Nutritional Risk Screening (NRS) 2002, a study in China estimated the prevalence of nutritional risk at 27.3% [7]. Results of a study in Britain using MUST as a screening tool reported a high prevalence of nutritional risk (19–65%) across patient groups [32]. A similar study in Israel employing MUST to screen patients suggested that 33% were at high nutritional risk [33].

The findings of our study are consistent with those reported in other clinical settings about the high prevalence of nutritional risk among adult inpatients [27, 31, 32]. Variations in prevalence of nutritional risk are attributed to the differing background characteristics in the patient population, pathology and screening tools applied [27]. For instance, the present study included different patients groups with various medical conditions admitted to medical and surgical wards. In addition, the study used MUST to determine nutritional risk among adult inpatients. However, MUST has been found to have excellent inter-rater reliability, concurrent and predictive validity [32, 34]. The extent of nutritional risk in the current study might have been underestimated due to the exclusion of critically-ill and older patients from the study. Nutritional risk has been reported to be higher among older patients due to physiological changes, loss of appetite, functional decline and socioeconomic factors [35].

Nutritional risk of hospitalized patients is associated with several factors such as patient’s health status, preadmission nutritional status and present pathology [27]. Other factors are related to physical, social and psychological dimensions of the patient. In order to mitigate the effects of malnutrition, it is important to identify nutritional risk based on predictive variables [27]. Identification of factors can facilitate attainment of patient’s nutritional goals and aid in the formulation of appropriate interventions [36]. Studies have specifically identified weight loss, appetite decrease, vomiting and dysphagia as some of the potential factors associated with nutritional risk [27, 36, 37].

Several attempts have been made by investigators to establish potential factors associated with nutritional risk using various statistical models [38]. Similar to our study, previous studies have used binary logistic regression to determine predictive variables of nutritional risk [39, 40]. Variables of sex, weight loss, appetite decrease, vomiting, diarrhoea and dysphagia investigated in this study have also been reported in other studies [27, 36, 37]. The current study reveals that vomiting, weakness, appetite decrease, dysphagia and weight loss were significantly associated (*p* < 0.05) with nutritional risk. This finding is consistent with other studies that have reported a significant association between weight loss, appetite decrease, vomiting, dysphagia and nutritional risk [27, 36, 37].

The observation that weight loss was the most significant factor related to nutritional risk (OR = 50.16, 95% CI = 5.75–36.70, *p* < 0.001) was also reported in a study conducted in South America [27]. The study observed that weight loss was the strongest predictor of nutritional risk (OR = 58.03, 95% CI: 18.46–182.41, *p* < 0.001) [27]. A similar study on nutritional risk among inpatients reported weight loss as the major predictor variable (OR = 37.7, 95% CI: 10.7–57.3, *p* < 0.001) [41]. Another study also reported that recent weight loss was associated with nutritional risk [1]. During hospitalization, many patients experience weight loss as a result of factors such as surgical procedures, disease effect and impaired appetite [42, 43]. Among patients, the process of weight loss demonstrates caloric depletion [42].

A study on the prevalence of nutritional risk reported that appetite decrease was significantly associated with nutritional risk (OR = 10.31, 95% CI: 2.23–47.55, *p* = 0.003) [27]. Several studies have pointed out that appetite decrease increases the likelihood of nutritional risk and possesses the best sensitivity and specificity [36, 37, 41]. Consistent with other studies, appetite decrease in our study increased the chance of nutritional risk by approximately 28.06 (OR = 28.06, 95% CI =1.49–8.12, *p* < 0.001) [37, 41]. Patient’s appetite could be impaired as a result of the disease condition, treatment and psychological factors [44]. Reduced appetite contributes to weight loss and adversely affects a patient’s nutritional status [27].

In the current study, vomiting and dysphagia were significantly associated with nutritional risk (*p* = 0.019, and *p* = 0.007 respectively). A similar study in Brazil found a significant association between vomiting, difficulties in swallowing and nutritional risk (*p* < 0.05) [27]. Studies suggest that variables associated with the digestive tract such as vomiting, dysphagia and diarrhoea are important risk factors [43, 45]. In contrast to the finding that diarrhoea is significantly associated with nutritional risk (*p* = 0.025), the current study reported otherwise [27]. In our study, diarrhoea was not a significant predictor of nutritional risk (*p* = 050). This is a surprising finding considering that there is a growing recognition that diarrhoea undermines nutritional status [46]. The possible explanation is that the current study involved different patient groups in both medical and surgical wards. Some studies have suggested that nutritional risk is relatively higher among patients in medical wards [47].

Malnutrition “is a state in which imbalance of energy, protein and other nutrients causes measurable adverse effects on body form and functionality” [26, 30]. Among the adverse consequences of malnutrition is muscle wasting and weakness resulting in impairment of respiratory and cardiac functionality [42]. Body weakness related to skeletal muscles delays return to mobility among hospitalized patients [42]. This study asked patients on the degree of weakness and results indicate that the variable was significantly associated with nutritional risk (OR = 7.07, 95% CI = 0.36–2.04, *p* = 0.008).

### Study limitations

The current study collected data from one teaching hospital in Zambia, therefore, results may not be generalized to all hospitals in the country. In spite of the limitation above, this study has generated useful information on nutritional risk and related factors that may be appropriated at the teaching hospital. Further studies preferably longitudinal in nature maybe warranted to collect more information to allow for more rigorous analysis of results.

## Conclusions

Findings of our study suggest that close to 60% of hospitalized patients at the teaching hospital are nutritionally at risk. Nutritional risk is an issue of major concern at the teaching hospital and is associated with a number of variables. Identification of nutritional risk using Malnutrition Universal Screening Tool among adult inpatients is feasible in resource-poor settings like ours. Weight loss and appetite decrease were the most significant factors associated with nutritional risk.

## Additional file

Additional file 1: Questionnaire used in the study. (DOC 31 kb)

## Abbreviations

ADA: American Dietetic Association
BMI: Body Mass Index
CI: Confidence interval
MoH: Ministry of Health
MUAC: Mid-upper arm circumference
MUST: Malnutrition Universal Screening Tool
NRS: Nutritional Risk Screening
OR: Odds Ratio
SD: Standard deviation
SPSS: Statistical Package for Social Sciences
TDRC: Tropical Disease Research Center
