# Supplementary material for: Nutritional risk and associated factors of adult in-patients at a teaching hospital in the Copperbelt province in Zambia; a hospital-based cross-sectional study
Source: BMC Nutr. 2018 Dec 6;4:40. doi: 10.1186/s40795-018-0249-4 (PMC7050804; doi:10.1186/s40795-018-0249-4)
Supplement: Supplementary file 1 — Questionnaire used in the study. (DOC 31 kb) [file 40795_2018_249_MOESM1_ESM.doc]

# Additional file 1: Questionnaire

**MALNUTRITION UNIVERSAL SCREENING TOOL (MUST) FOR ADULT IN-PATIENTS**

**Name of Ward:_____________________________Date____/____/________ Responsible officer:___________**

**Section A: Answer all questions related to this section to determine the Body Mass Index (BMI)**

1A. Weight in Kilograms (Kg) = ______________________

2A. Ulna length (cm) = ______________________________

3A. Estimated height (cm) = __________________________

4A. Body Mass Index = ______________________________ Kg/m^2^

**Section B: Answer all questions related to Malnutrition Universal Screening Tool (MUST) in the table below.**

| **Bed Number** | **Age** | **Sex** | **Recent hospitalization** | **Diarrhoea** | **Vomiting** | **Weakness** | **Appetite decrease** | **Dysphagia** | 1. **MUAC (cm)** | **Estimate nutritional status based on physical appearance.** | **Determine unplanned weight loss (%) in the past 3 – 6 months.** | **Estimate BMI Category based on findings (1), (2) & (3)** | **Consider acute disease effect** | **Overall score**  **0 = Low risk**  **1=Medium risk**  **2=High risk** |
| --- | --- | --- | --- | --- | --- | --- | --- | --- | --- | --- | --- | --- | --- | --- |
|  |  | M/  F | Yes  /  No | Yes  /  No | Yes  /  No | Yes  /  No | Yes  /  No | Yes  /  No |  | **Obvious wasting = 4**  **Moderate wasting = 3**  **Acceptable weight = 2 Overweight =1**  **Very Overweight =0** | < 5% = **0**  5 – 10% = **1**  > 10% = **2** | BMI > 20 = **0**  BMI 18.5 – 20 = **1**  BMI <18.5= **2** | If patient is acutely ill and there has been little or is likely to be no nutritional intake for > 5 days, score = **2,**  **if not score=0** |  |
|  |  |  |  |  |  |  |  |  |  |  |  |  |  |  |
